# A cryptic oxidoreductase safeguards oxidative protein folding in *Corynebacterium diphtheriae*

**DOI:** 10.1073/pnas.2208675120

**Published:** 2023-02-14

**Authors:** Melissa E. Reardon-Robinson, Minh Tan Nguyen, Belkys C. Sanchez, Jerzy Osipiuk, Christian Rückert, Chungyu Chang, Bo Chen, Rahul Nagvekar, Andrzej Joachimiak, Andreas Tauch, Asis Das, Hung Ton-That

**Affiliations:** ^a^Department of Microbiology & Molecular Genetics, University of Texas McGovern Medical School, Houston, TX 77030; ^b^Division of Oral and Systemic Health Sciences, School of Dentistry, University of California, Los Angeles, CA 90095; ^c^Department of Pathology and Immunology, Baylor College of Medicine, Houston, TX 77030; ^d^Center for Structural Genomics of Infectious Diseases, Consortium for Advanced Science and Engineering, University of Chicago, Chicago, IL 60637; ^e^Structural Biology Center, Argonne National Laboratory, Lemont, IL 60439; ^f^Center for Biotechnology, Bielefeld University, D-33615 Bielefeld, Germany; ^g^Stanford University, Stanford, CA 94305; ^h^Department of Medicine, Neag Comprehensive Cancer Center, University of Connecticut Health Center, Farmington, CT 06030; ^i^Molecular Biology Institute, University of California, Los Angeles, CA 90095; ^j^Department of Microbiology, Immunology & Molecular Genetics, University of California, Los Angeles, CA 90095

**Keywords:** *Corynebacterium diphtheriae*, disulfide bond, pili, diphtheria toxin, gram-positive bacteria

## Abstract

In many gram-positive Actinobacteria, including *Actinomyces oris* and *Corynebacterium matruchotii*, the conserved thiol-disulfide oxidoreductase MdbA that catalyzes oxidative folding of exported proteins is essential for bacterial viability by an unidentified mechanism. Intriguingly, in *Corynebacterium diphtheriae*, the deletion of *mdbA* blocks cell growth only at 37 °C but not at 30 °C, suggesting the presence of alternative oxidoreductase enzyme(s). By isolating spontaneous thermotolerant revertants of the *mdbA* mutant at 37 °C, we obtained genetic suppressors, all mapped to a single T-to-G mutation within the promoter region of *tsdA*, causing its elevated expression. Strikingly, increased expression of *tsdA*—via suppressor mutations or a constitutive promoter—rescues the pilus assembly and toxin production defects of this mutant, hence compensating for the loss of *mdbA*. Structural, genetic, and biochemical analyses demonstrated TsdA is a membrane-tethered thiol-disulfide oxidoreductase with a conserved CxxC motif that can substitute for MdbA in mediating oxidative folding of pilin and toxin substrates. Together with our observation that *tsdA* expression is upregulated at nonpermissive temperature (40 °C) in wild-type cells, we posit that TsdA has evolved as a compensatory thiol-disulfide oxidoreductase that safeguards oxidative protein folding in *C. diphtheriae* against thermal stress.

## Significance

Oxidative protein folding via disulfide bond formation is an important process in bacteria, although it can be dispensable in various organisms. In many gram-positive Actinobacteria, deletion of *mdbA* coding for the conserved thiol-disulfide oxidoreductase MdbA that catalyzes oxidative folding of exported proteins is lethal for cell growth by an uncharacterized mechanism. However, *Corynebacterium diphtheriae* cells lacking *mdbA* are viable at 30 °C, suggesting the presence of alternative oxidoreductase(s) recompensing the loss of *mdbA*. Using genetic suppressor, structural, and biochemical analyses, we provide evidence to support that *C. diphtheriae* encodes TsdA as a compensatory thiol-disulfide oxidoreductase safeguarding oxidative protein folding in this actinobacterium against thermal stress. This study expands our understanding of oxidative protein folding mechanisms in the understudied Actinobacteria.

Both eukaryotes and prokaryotes, including fungi, archaea, and bacteria, possess a large family of thioredoxin-like proteins that catalyze oxidative protein folding via disulfide bond (Dsb) formation (1, 2). In the endoplasmic reticulum (ER) of eukaryotes, protein disulfide isomerases (PDIs) play an important role in this folding process (3). PDI activity was first reported by Anfinsen and colleagues in a rat liver system that reactivates reduced ribonuclease (RNase) and lysozyme as substrates (4). In the periplasm of gram-negative bacteria, the well-studied *Escherichia coli* DsbA and DsbB proteins constitute a Dsb-forming complex, with the periplasmic thiol-disulfide oxidoreductase DsbA catalyzing Dsb formation in cysteine-containing substrates (5) while the cytoplasmic membrane partner DsbB acts to reactivate DsbA (6, 7). DsbB, in turn, needs to be reoxidized, a process that involves quinone – a component of the bacterial electron transport chain (ETC) (8). Like PDIs, both DsbA/DsbB enzymes harbor a catalytic CxxC motif that is essential for thiol-disulfide exchange reactions (9, 10). Intriguingly, although DsbA homologs are found in many gram-positive Firmicutes, these bacteria largely exclude cysteine residues in their cytoplasmic and exported proteins, especially in aerobic species with 70% of exported proteins devoid of cysteines (11). In contrast, gram-positive Actinobacteria, such as *Actinomyces oris* and *Corynebacterium diphtheriae*, export a variety of proteins, roughly 60% of which contain even numbers of cysteine residues (11, 12), suggesting that oxidative protein folding is a major physiological pathway in these organisms.

Indeed, the membrane-tethered thiol-disulfide oxidoreductase MdbA of *A. oris* is a major protein folding machine that catalyzes Dsb formation in pilins, which form covalently linked polymers that are essential for polymicrobial interactions and biofilm formation (13). Deletion of *A. oris mdbA* is lethal (13), indicating that the substrates of MdbA extend beyond pilins and that they are involved in central cellular processes, including cell division and cell wall biosynthesis (13). Once MdbA catalyzes a thiol-disulfide exchange reaction, it needs to be reactivated; reactivation of MdbA was found to require a vitamin K epoxide reductase (VKOR), which functions like a DsbB counterpart (13). Notably, rejuvenation of the MdbA/VKOR system is driven by the NADH (nicotinamide adenine dinucleotide + hydrogen) dehydrogenase and menaquinone biosynthesis of the *A. oris* ETC (14). Similar to *A. oris*, the Actinobacterium *C. diphtheriae* possesses a homolog of MdbA (less than 22% identity), whose crystal structure harbors a thioredoxin-like fold, an extended α-helical domain, and a CxxC motif in its active site (15), which are common features of Actinobacterial thiol-disulfide oxidoreductases, including *A. oris* and *Corynebacterium matruchotii* MdbA enzymes (15–18). Importantly, it remains unclear how *C. diphtheriae* MdbA is reactivated, despite efforts of generating mutants of genes coding for putative DsbB-like proteins and oxidoreductases, such as DIP0397 and DIP0411, which do not exhibit any defective phenotypes (15), and no VKOR homologs are found in *C. diphtheriae*. Because *C. diphtheriae* MdbA is required for growth, adhesive pilus assembly, diphtheria toxin (DT) production, and virulence, MdbA is believed to have broad substrate specificity and constitute a major Dsb-forming machine in this organism (15). Like *A. oris* and *C. matruchotii*, deletion of *C. diphtheriae mdbA* is lethal at 37 °C (13, 18); however, the *C. diphtheriae mdbA* mutant is viable at 30 °C (15). This conditional lethality of the deletion mutant at high temperature raised the possibility that an additional oxidoreductase enzyme present in the organism might compensate for the loss of MdbA when cells are in a lower metabolic demand.

To test our hypothesis, we adopted a classic suppressor screen and selected thermotolerant revertants of the *C. diphtheriae mdbA* mutant. The characterization of these revertants enabled us to unveil here a functional homolog, TsdA (formerly DIP0397 as mentioned above), whose expression was upregulated in three independent suppressor mutants, as a thiol-disulfide oxidoreductase capable of replacing MdbA and mediating Dsb formation in both pilin and DT as model substrates. Given our finding that *tsdA* expression is upregulated under heat stress, we propose that TsdA is a stress-adaptive thiol-disulfide oxidoreductase that protects corynebacteria under stress conditions.

## Results

### Identification of Compensatory Mutants for *mdbA* Deletion in *C. diphtheria*.

**Fig. 1.**
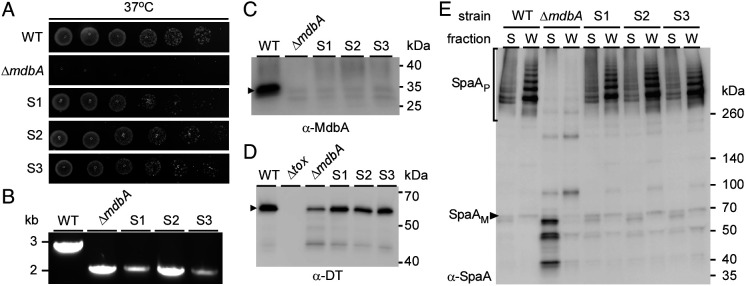
Phenotypic Characterization of Genetic Suppressors of the *C. diphtheriae* Δ*mdbA*. (*A*) Cultures of *C. diphtheriae* strains, including three suppressor mutants S1–3, grown at 30 °C to saturation were tested for growth at higher temperature by spotting aliquots of serial dilutions (10^–3^ to 10^–8^ dilution) on agar plates and overnight incubation at 37 °C. (*B*) Chromosomal DNA isolated from indicated strains was used to PCR-amplify 1-kB regions up- and downstream of *mdbA*. (*C*) Protein samples isolated from membrane fractions of *C. diphtheriae* strains grown at 30 °C were analyzed by SDS-PAGE and immunoblotted with antibodies against MdbA (α-MdbA); predicted MW of 27 kDa. (*D*) DT collected from the culture supernatant of indicated strains grown at 30 °C and treated with iron chelators was analyzed by SDS-PAGE and immunoblotted with antibodies against DT (α-DT); predicted MW of 61 kDa. (*E*) Protein samples isolated from the culture supernatant (S) and cell wall (W) fractions of indicated *C. diphtheriae* strains were analyzed by SDS-PAGE using 4 to 20% gradient gels and immunoblotted with antibodies against SpaA (α-SpaA). SpaA monomers (M) and polymers (P) are indicated.

Because the *mdbA* mutant (Δ*mdbA*) of *C. diphtheriae* is viable at 30 °C (15), we considered that an additional oxidoreductase with suboptimal expression or activity might compensate for the loss of MdbA under a low metabolic rate, for example at 30 °C. To test this, we asked whether revertants could be isolated at high temperature in which a suppressor mutation might elevate expression or activity of a potential homolog so as to compensate for the missing function of MdbA. Indeed, when stationary-phase cultures of the *mdbA* mutant grown at 30 °C were diluted in fresh cultures and grown overnight at 37 °C, and then plated for survivors at 37 °C, three surviving colonies (referred to as suppressors S1, S2, and S3) were recovered. In subsequent tests, these suppressors retained the thermotolerant phenotype and grew like wild-type cells at the nonpermissive temperature (Fig. 1*A*). PCR amplification of the *mdbA* gene from chromosomal DNA confirmed the absence of *mdbA* in these isolates, like the *mdbA* mutant (Fig. 1*B*). Likewise, immunoblotting with antibodies against MdbA (α-MdbA) also demonstrated the absence of membrane-bound MdbA in all three suppressors as in the *mdbA* mutant, in contrast to the wild-type cells that showed the presence of MdbA (Fig. 1*C*).

Since DT and pilins contain Dsbs that are required for proper protein folding (19–21) and are the experimentally documented substrates of MdbA (15), we analyzed the cell culture medium and cell wall of the three suppressors S1, S2, and S3 for the presence of these proteins by immunoblotting as previously reported (15). Compared to the *mdbA* mutant, each suppressor displayed an increased level of the exotoxin DT that was nearly comparable to the wild-type (Fig. 1*D*). Remarkably, pilus polymerization and cell wall anchoring of SpaA pili were also restored in the three suppressors, contrary to their gross defects observed in the *mdbA* mutant (Fig. 1*E*). Furthermore, by electron microscopy cells of the three suppressors exhibited normal cell morphology like the wild-type cells, in contrast to the *mdbA* mutant, which displayed chained and coccoid morphology at nonpermissive temperature (*SI Appendix*, Fig. S1), a phenotype indicative of cell division defects as previously reported (15). The results suggest that all three suppressors might harbor compensatory mutations that rescued MdbA-mediated oxidative protein folding.

### A Compensatory Mutation Resulting in Elevated Expression of the Thiol-Disulfide Oxidoreductase TsdA Is Responsible for Suppression of mdbA Deletion.

**Fig. 2.**
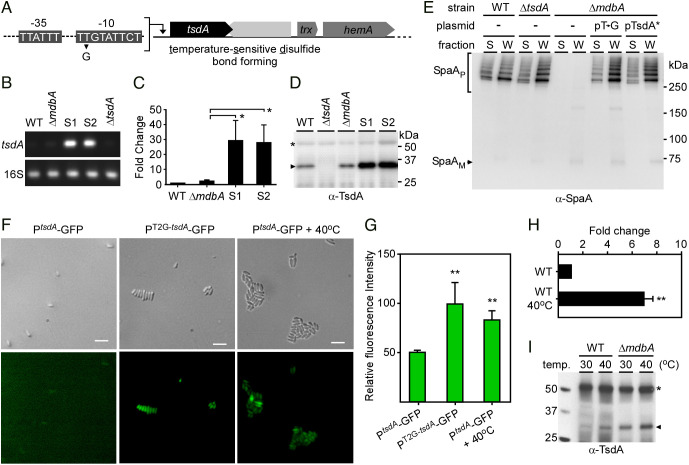
Overexpression of the Thiol-Disulfide Oxidoreductase TsdA Suppresses Lethality of *mdbA* Deletion at Nonpermissive Temperature. (*A*) Whole-genome sequencing of three suppressor mutants S1, S2, and S3 revealed a single T-to-G substitution in the −10 region upstream of a thiol-disulfide oxidoreductase-encoding gene, *tsdA*, with a gene coding for a HAD-IB family hydrolase downstream of *tsdA* shown in light gray. (*B* and *C*) The *tsdA* transcript level was determined by qRT-PCR, with *tsdA* expression in different strains, relative to the WT, normalized to 16S rRNA levels. Results are presented as average of three independent experiments performed in triplicate, with significance analyzed by an unpaired *t* test using GraphPad. (*D*) Protein samples from the membrane fractions of indicated *C. diphtheriae* strains were analyzed by immunoblotting with antibodies against TsdA (α-TsdA); predicted MW of 31 kDa. A nonspecific band migrating above the 50-kDa marker was used as loading control (*). (*E*) Protein samples isolated from the culture supernatant and cell wall fractions of indicated *C. diphtheriae* strains were analyzed by immunoblotting with α-SpaA. Polymeric (P) and monomeric (M) forms of SpaA are indicated. Of note, pT→G and pTsdA* are plasmids expressing *tsdA* under the control of either the *tsdA* promoter with T-to-G substitution or the AraC regulator protein, respectively. (*F *and *G*) Cells of a *tsdA* mutant harboring a plasmid expressing GFP under the control of the native *tsdA* promoter or this promoter with T-to-G substitution were analyzed by fluorescence microscopy at 30 °C or 40 °C; (Scale bar indicates 5 μm.) Relative fluorescence intensity was measured using a microplate reader, with error bars representing SDs from biological triplicates. (*H*) Expression of *tsdA* was determined by qRT-PCR. Average values of two experiments performed in triplicate are shown. **P < 0.005 and ***P < 0.0001 were determined by an unpaired two-tailed *t* test using GraphPad Prism. (*I*) Aliquots of equivalent cultures of indicated strains grown at 30 °C were inoculated at 30 °C or 40 °C for 30 min prior to harvesting of membrane fractions for immunoblotting with a-TsdA as described in D.

To characterize genetic alterations that compensate for the loss of *mdbA* in three aforementioned suppressors, we performed whole genome sequencing, which revealed a single T-to-G nucleotide substitution in all three chromosomal samples. This mutation was found within a promoter region upstream of a putative membrane-bound thiol-disulfide oxidoreductase-encoding gene, DIP0397, hereby referred to as *tsdA* (tsd for temperature-sensitive Dsb forming) (Fig. 2*A*). It is important to recall that we previously reported that deletion of DIP0397 produced no detectable phenotypes as compared to the wild-type strain (also see Fig. 2*E*) (15). Furthermore, the isolate S2 contains an Arg-to-His mutation (R62H) within a predicted metallohydrolase (DIP0104), whereas S3 was identical to S1; therefore, S1 and S2 were used for further characterizations.

To further characterize the effects of the T-to-G mutation at the putative promoter of *tsdA*, we identified the transcriptional start site (TSS) of *tsdA* by implementing Rapid Amplification of cDNA Ends (5′-RACE) with total RNA samples isolated from the wild-type and S1 strains. 5′-RACE PCR products were analyzed by Sanger sequencing, revealing the same TSS of *tsdA* in both samples located at the 15th nucleotide upstream of the start codon Adenine-Uracil-Guanine (AUG) (*SI Appendix*, Fig. S2). This is consistent with our previous findings using RNA-seq analysis (22). Based on the identified *tsdA* TSS, the upstream DNA region was analyzed for conserved promoter motifs, and a promoter structure was deduced, in which the T-to-G substitution in all suppressors occurred at the potential -10 region of the *tsdA* promoter Fig. 2*A*.

To examine whether the T-to-G mutation affects transcription of *tsdA*, we performed qRT-PCR and found that *tsdA* expression in S1 and S2 was approximately 30 times higher than the wild-type and Δ*mdbA* mutant strains grown at 30 °C (Fig. 2 *B* and *C*). In addition, immunoblotting with antibodies against TsdA (α-TsdA) showed increased expression of TsdA in S1 and S2, as compared to the wild-type and Δ*mdbA* mutant strains (Fig. 2*D*). These results indicate that the increased expression of TsdA, due to T-to-G substitution, might rescue the defects of Δ*mdbA*. To determine whether this is the case, we generated plasmids expressing *tsdA* from its native promoter region in the isolate S1 with an identical T-to-G mutation (pT_→_G) or a constitutive promoter (pTsdA*) and electroporated them into the Δ*mdbA* mutant. By immunoblotting of protein samples isolated from culture medium (M) and cell wall (W) fractions of these strains, we observed that the Δ*mdbA* mutant harboring pT_→_G or pTsdA* produced SpaA polymers at a level similar to the wild-type strain (Fig. 2*E*). Note that a mutant lacking *tsdA* assembled the wild-type level of pilus polymers (Fig. 2*E*), demonstrating the sufficiency of MdbA in Dsb formation, and we failed to generate a double mutant lacking both *tsdA* and *mdbA* after several attempts, confirming the essentiality of *mdbA*.

Given that the suppressors S1, S2, and S3 are thermotolerant as opposed to the Δ*mdbA* mutant, we sought to determine if temperature elevation affects *tsdA* expression. For convenient monitoring of *tsdA* expression, we constructed two *gfp* transcriptional fusion plasmids, in which expression of GFP is driven by either the native *tsdA* promoter (p*^tsdA^*) or the *tsdA* promoter with T-to-G mutation (p^T2G-^*^tsdA^*). Each vector was electroporated into the *tsdA* mutant and promoter activity was determined by fluorescence microscopy. As expected, cells expressing GFP from the p^T2G-^*^tsdA^* promoter showed significantly high signal of GFP fluorescence, in contrast to cells expressing GFP from the wild-type *tsdA* promoter (Fig. 2 *F* and *G*). Strikingly, increased fluorescence was observed when the latter cells with the native *tsdA* promoter were exposed to heat (40 °C) for 30 min (Fig. 2 *F* and *G*). Consistent with these results, qRT-PCR analysis demonstrated that *tsdA* expression in wild-type cells was induced nearly eightfold after heat treatment (Fig. 2*H*), and western blotting analysis showed increased production of TsdA at 40 °C (Fig. 2*I*). At this temperature, overexpression of *tsdA* in the *mdbA* mutant rescued its pilus assembly defect at the same level as seen at 30 °C (*SI Appendix*, Fig. S3). These results suggest that TsdA may function as a compensatory thiol-disulfide oxidoreductase under heat stress conditions.

### Structural Determination of TsdA Reveals a Thiol-Disulfide Oxidoreductase Fold.

**Fig. 3.**
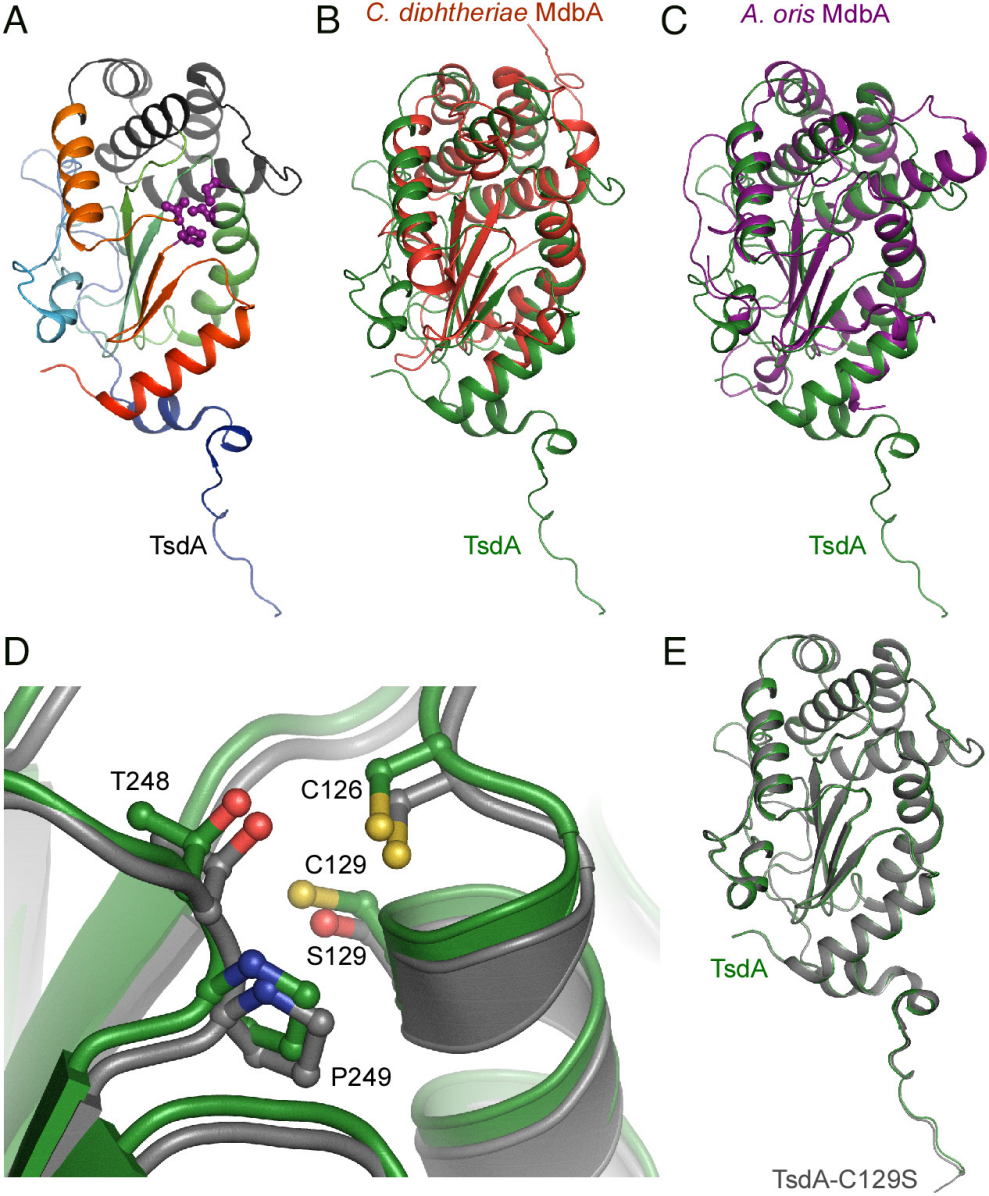
Crystal Structures of the *C. diphtheriae* Thiol-Disulfide Oxidoreductase TsdA and Its C129S Variant. (*A*) Shown is an overall structure of the *C. diphtheriae* TsdA determined by X-ray crystallography. Rainbow colors from blue to red indicate the positions of N-terminal and C-terminal residues. The α-helical domain (residues 161 to 229) is shown in gray. Active site residues, C126, C129, T248 and P249, are shown as ball-and-sticks in magenta. (*B*, *C*, and *E*) Structure alignments of *C. diphtheriae* TsdA (green) with *C. diphtheriae* MdbA (red; PDB:5C00), *A. oris* MdbA (purple; PDB:4Z7X), and TsdA-C129S mutant (gray) are shown. (*D*) The active centers of *C. diphtheriae* TsdA and its C129S mutant are superimposed, with the wild-type structure shifted upward after superimposition for clarity.

That the overexpression of *tsdA* can rescue the pleotropic defect of *C. diphtheriae* cells devoid of the primary thiol-disulfide oxidoreductase MdbA suggested that TsdA has Dsb-forming capability. To reveal the structural basis for the potential thiol-disulfide oxidoreductase activity of TsdA, we determined the *C. diphtheriae* wild-type TsdA structure by X-ray crystallography refracted to 1.45 Å atomic resolution with R-work and R-free factors equal to 11.4 and 15.3 %, respectively (*SI Appendix*, Tables S1 and S2). The structure is a single continuous amino acid chain covering 38 to 282 residues of the protein (full length MW of 31 kDa). Despite using a recombinant protein lacking its transmembrane domain (1–32) for crystallization, four N-terminal and seven C-terminal residues are not visible in the structure, most likely due to flexibility of these fragments. The overall structure represents a typical DsbA/MdbA protein family fold (13, 16, 17), including a thioredoxin-like domain and an α-helical domain, which are found in actinobacterial MdbA proteins (13, 15, 18) (Fig. 3 *A*–*C*). The thioredoxin-like domain (residues 54 to 160 and 230 to 282) consists of a five-strand β-sheet in an order of β1↑- β3↓- β2↓- β4↑- β5↓ and six flanking helices (one 3_10_-helix, ƞ1, and five α-helices, α2-4 and α9-10). The thioredoxin-like domain is separated by the amino acid segment 161 to 229 of the α-helical domain. The conserved catalytic CPFC motif, residues 126 to 129, forms the active site together with a conserved cis-Pro loop (residues T248 and P249) (Fig. 3*D*). The C126 residue is located at the N-terminal end of the kinked α-helix 126 to 136; such a location of the CXXC motif is characteristic of DsbA proteins (16, 17, 23). The cysteine sulfurs in the CXXC motif are 3.40 Å apart with clear separation of corresponding electron densities; thus, the structure represents a reduced form of the protein. The CXXC motif and the cis-Pro loop in TsdA bear close resemblance to those of other DsbA proteins that participate in substrate binding (24, 25). In addition, the α-helical domain of TsdA is comprised of four α-helices. The N-terminal end of the protein (residues 38 to 53 of the structure) is nearly unstructured with a short α1 helix. This fragment is visible due to stabilization by two neighbor protein molecules in crystals. Of note, no additional cysteine residue is found in TsdA.

The TsdA structure very closely resembles the TsdA-C129S protein structure, obtained from the same crystallization conditions, and diffracted to 1.10 Å atomic resolution with R-work and R-free factors equal to 11.6 and 13.4%, respectively (*SI Appendix*, Tables S1 and S2) (Fig. 3*E*). Ser129 mimics exactly the catalytic Cys129 conformation in the wild-type structure (Fig. 3*D*). The rest of the protein is principally the same in both structures with RMSD (rmsd of superimposed Cα atoms equal to 0.183 Å. When searching for structural homologs of TsdA, we noticed that our TsdA structure is fundamentally similar to a structure of the same protein previously named DsbA (PDB:4PWO) by Um et al. (26) (*SI Appendix*, Fig. S4*A*), with RMSD equal to 0.760 Å for 234 aligned residues. The active site configuration is exactly the same as the one in our wild-type TsdA protein structure. The main difference between our structures and 4PWO is the positioning of the N-terminus, which is moved farther away from the protein main body by 2 to 4 Å (*SI Appendix*, Fig. S4*A*). There are also slight changes in conformation of 66 to 77 and 245 to 247 loops; probably resulted from slightly different crystallization conditions and crystal packing, these differences are unlikely to have any biological impact.

Evidently, the TsdA structure has features characteristic of the DsbA family proteins(16, 17) (*SI Appendix*, Fig. S4 *B* and *C*). According to DALI analysis(27), the closest TsdA structural homolog is the *Bacillus subtilis* oxidoreductase BdbD despite of low amino acid identity at 15.4% (PDB:3EU3) (*SI Appendix*, Fig. S4*C*). While BdbD contains a novel metal site (25), in TsdA, no calcium binding site is found. The major difference between TsdA and BdbB structures is the N-terminal part of the enzymes, which is significantly shorter in the case of BdbD (*SI Appendix*, Fig. S4*C*). The alignment of those structures has a Z score and RMSD equal to 20.8 and 2.0, respectively, for 186 equivalent residues. The next closest structural homologs are *Silicibacter pomeroyi* DSS-3 protein (PDP:3GYK) and *Wolbachia pipientis* thiol-disulfide exchange protein DsbA2(28) (PDB:6EEZ) having Z scores of 19.2 and 18.5 and RMSDs of 2.4 and 2.6, respectively.

### TsdA Possesses Thiol-Disulfide Oxidoreductase Activity That Requires the Conserved CxxC Motif.

**Fig. 4.**
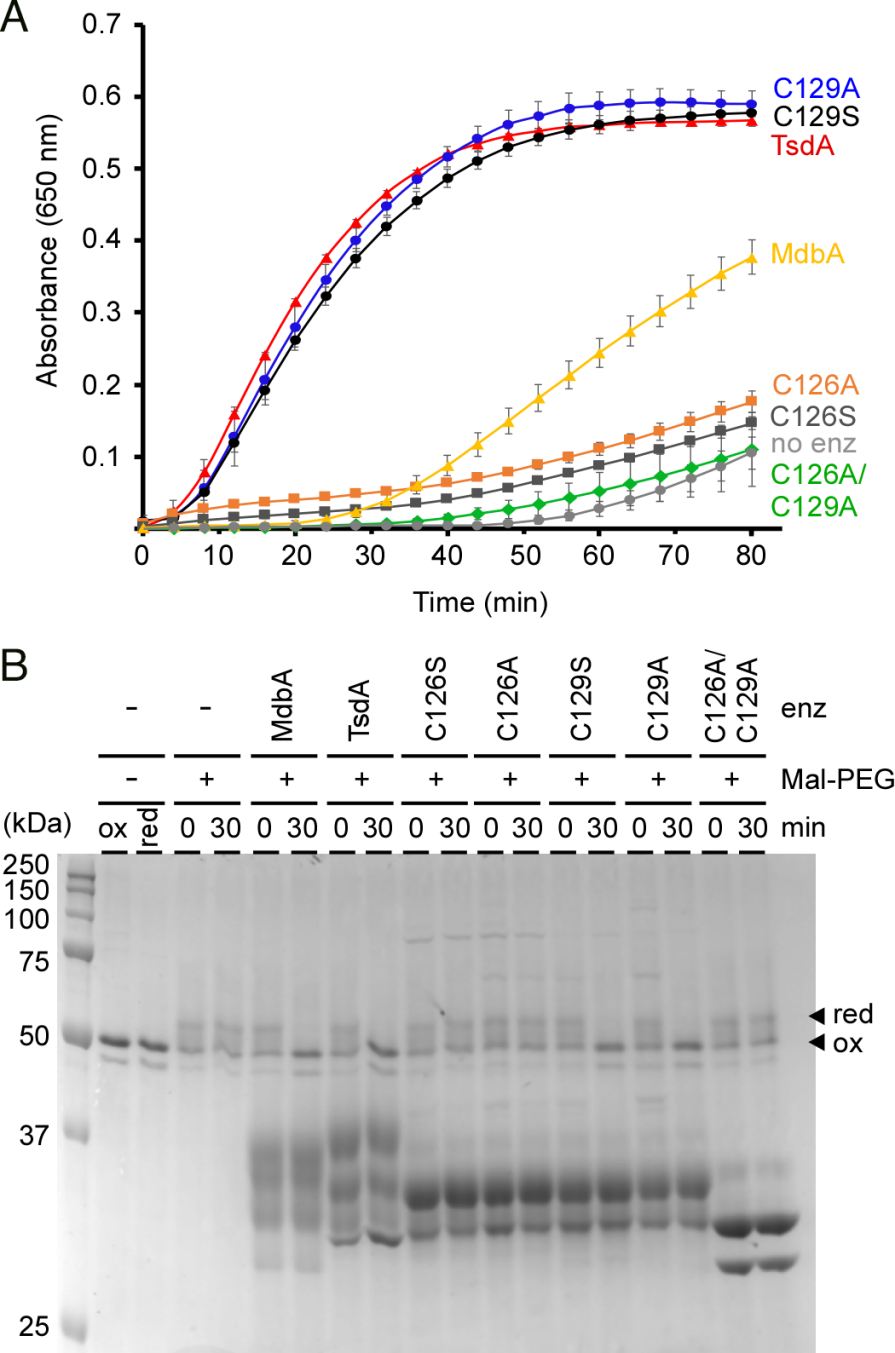
Thiol-Disulfide Oxidoreductase Activity of TsdA Involves the Catalytic CXXC Motif. (*A*) Insulin (170 µM) was used in reactions containing 0.5 mM DTT, 2 mM EDTA, 30 µM purified enzymes (MdbA or TsdA), and 100 mM potassium acetate buffer, pH 7.5, and were incubated at 25 °C. Insulin turbidity was monitored at 650 nm every 4 min for 80 min. Data are presented as average from three independent experiments performed in triplicate. Significance was analyzed by Dunnett's post hoc test using GraphPad Prism; ns, not significant and ***, *P* < 0.0001. (*B*) Reduced FimA was treated with purified MdbA (22 kDa), TsdA (27 kDa), TsdA mutants, or no (lanes -) enzymes in a redox buffer system (100 mm Tris-HCl, pH 4.0, 2 mm EDTA, 0.2 mm GSSG, 1 mm GSH). At indicated times, the reactions were quenched by Mal-PEG, and protein samples were analyzed by SDS-PAGE with Coomassie staining. Reduced and oxidized forms of FimA are indicated by arrowheads. Note, there is no change in migration of these forms without Mal-PEG.

Considering previous studies demonstrating that the so-called *C. diphtheriae* DsbA, or now TsdA, may possess Dsb isomerase/reductase activity in vitro using RNase A as a substrate (26) and our results presented above, we surmised that TsdA is a thiol-disulfide oxidoreductase capable of replacing the major oxidative folding machine MdbA via the conserved catalytic CPFC motif. We first examined whether TsdA has enzymatic reduction activity by generating recombinant wild-type TsdA and its variants with the conserved cysteine residues mutated to Ser or Ala using an insulin reduction assay (29). As shown in Fig. 4*A*, wild-type TsdA catalyzed reduction of insulin leading to cleavage of two interchain Dsbs, resulting in precipitation of the insulin ß-chain, which was measured at 650 nm. Remarkably, the reduction activity of TsdA was greater than that of MdbA and required the conserved C126 residue as mutation of this residue, but not C129, abrogated the enzymatic activity (Fig. 4*A*), while neither mutation affected the stability of the recombinant proteins (*SI Appendix*, Fig. S5). As expected, a TsdA mutant with both cysteines mutated to alanine failed to reduce insulin (Fig. 4*A*).

Since MdbA catalyzes Dsb formation in pilin substrates (13, 15, 18), we used the same set of enzymes in a Dsb reconstitution assay with the pilin FimA as substrate (13), in which reduced FimA was treated with TsdA enzymes for 30 min, followed by treatment with the sulfhydryl-reactive reagent methoxypolyethylene glycol-maleimide (Mal-PEG)—which adds 2-kDa molecular mass to reduced FimA—and SDS-PAGE electrophoresis. Like MdbA, wild-type TsdA catalyzed Dsb formation in FimA, leading to an oxidized form migrating faster on SDS-PAGE than a reduced form (Fig. 4*B*). Consistent with the above results, mutations of C126 or both C126 and C129 abrogated enzymatic activity, whereas mutations of C129 did not (Fig. 4*B*).

**Fig. 5.**
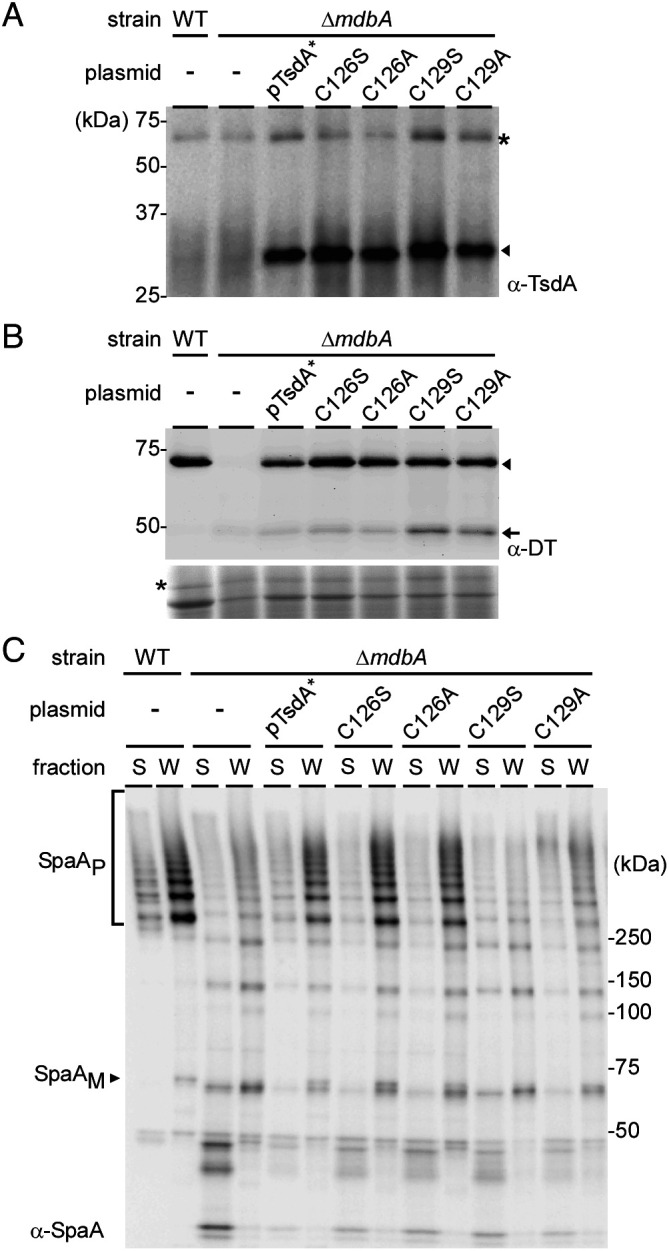
Replacement of MdbA by TsdA in vivo requires the catalytic CXXC motif. Cells of indicated *C. diphtheriae* strains grown at 30 °C to mid-log phase were subjected to cell fractionation. Protein samples in the membrane (*A*), culture supernatant (*B*), or culture supernatant and cell wall (*C*) fractions were immunoblotted with α-TsdA (31 kDa), α-DT (61 kDa) or α-SpaA (53 kDa), respectively. Intact DT and its degradation product in *B* are marked by an arrowhead and an arrow, respectively. Nonspecific bands (*) were used as loading control.

Because overexpression of wild-type TsdA in the Δ*mdbA* strain rescued the defects in SpaA pilus assembly and DT production by this mutant (Figs. 1 and 2), we examined whether mutations of the CxxC motif also affect the oxidative folding activity of TsdA in vivo by expressing individual TsdA variants in the Δ*mdbA* mutant. Critically, mutations of the CxxC motif did not affect TsdA expression or stability in vivo (Fig. 5*A*). While expression of wild-type TsdA or TsdA with C126 mutations in the Δ*mdbA* mutant restored DT expression, expression of TsdA with C129 mutations caused degradation of DT resulting in an increased level of a fast-migrating species (Fig. 5*B*). Similarly, in the pilus assembly assay as described in Fig. 2*E*, mutations of C129 hindered pilus assembly, whereas C126 mutations did not show any defect in pilus assembly compared to the WT strain and wild-type TsdA (Fig. 5*C*). Clearly, these results altogether demonstrate that TsdA is a potent thiol-disulfide oxidoreductase that when expressed at a sufficient level is capable of replacing the major oxidative protein-folding enzyme MdbA in *C. diphtheriae*, and that its enzymatic activity requires the conserved catalytic CxxC motif.

## Discussion

Dsbs are important for the proper structural folding, stability, and function of many bacterial proteins exported to the extracytoplasmic compartment through the Sec translocon. Within gram-negative bacteria, these disulfide linkages are catalyzed in the periplasm by the thiol-disulfide oxidoreductase pair DsbA and DsbB, while the Dsb isomerization system DsbC/DsbD is present as the repair machinery to reduce and isomerize any misformed Dsbs (30). In gram-positive Actinobacteria like the oral commensal/pathogens *C. diphtheriae* and *A. oris*, the thiol-disulfide oxidoreductase MdbA is a major Dsb-forming machine that catalyzes post-translocational folding of exported proteins(12). Unlike *E. coli dsbA*, which is dispensable as a function, although indispensable in anaerobic conditions, *mdbA* is an essential gene in Actinobacteria. A striking variation in this theme is that the *C. diphtheriae mdbA* mutant, although inviable at 37 °C, forms colonies at 30 °C (15), which led to our speculation that *C. diphtheriae* might encode an additional thiol-disulfide oxidoreductase that can replace MdbA. We reveal here the identity and characteristics of this thiol-disulfide oxidoreductase, TsdA, as a potent compensatory enzyme that appears to safeguard oxidative protein folding in *C. diphtheriae* under stress conditions.

The conditional lethality of the *mdbA* mutant of *C. diphtheriae* provided a powerful tool to identify suppressor mutants that survive elevated temperature despite the genetic depletion of *mdbA*. We isolated and characterized three revertants forming colonies at the nonpermissive temperature and found a single-point mutation within the predicted −10 box of the *tsdA* promoter in each of these isolates (Figs. 1 and 2). Subsequently, we demonstrated that this mutation causes increased expression of *tsdA*, thereby restoring Dsb formation in the *mdbA* mutant (Fig. 2). Though we did not investigate the mechanism by which this mutation alters *tsdA* expression, it is logical to infer that it extends and optimizes the -10 box to increase the basal rate of transcription initiation. Extended -10 boxes are alternative δ^70^-type promoter elements commonly found in gram-positive bacteria (31–34). The extended -10 box, which features a TRTGNTATAAT consensus sequence, harbors a TG dinucleotide that lowers the thermal energy required to form an open RNA polymerase initiation complex (31). The -10 box within the wild-type *tsdA* promoter (TTTTGTATTCT) is similar to the extended sequence, but it lacks the TG dinucleotide at the proper position. Thus, the T-to-G substitution within the promoter might create this properly placed TG element (TTTGGTATTCT), resulting in higher levels of *tsdA* transcription than the wild-type.

Is TsdA important for Dsb formation and proper folding of secreted proteins in Corynebacteria? Although we were able to delete *tsdA* in *C. diphtheriae* containing *mdbA*, demonstrating dispensability of TsdA function, our multiple attempts to construct a Δ*tsdA* Δ*mdbA* double mutant were unsuccessful. Thus, the basal expression of *tsdA* is required to maintain the viability of the *mdbA* mutant at 30 °C. The failure to generate the double t*sdA*/*mdbA* mutant also suggests that TsdA may be the only factor that can substitute MdbA. Consistent with this scenario, overexpression of *tsdA* in the *mdbA* mutant by reconstituting the vector-borne T-to-G mutation or using an arabinose-inducible promoter in the *mdbA* mutant rescues the defects in pilus assembly and DT production (Fig. 2). The interchangeability of TsdA to MdbA implies that TsdA is a thiol-disulfide oxidoreductase. Indeed, our own structural determination of TsdA and previous data (26) reveal the very features of TsdA that are also found in MdbA structures (13, 15, 18): a thioredoxin-like domain, an α-helical domain, which shares with canonical DsbA proteins, and a catalytic CxxC motif as part of a dipole at the end of the α-helix that is thought to account for the reversible oxidation of the CXXC motif as in DsbA (24, 35) (Fig. 3). In fact, the CxxC motif is required for the thiol-disulfide oxidoreductase activity of TsdA that is central to pilus assembly and toxin production in the absence of MdbA (Figs. 4 and 5). Mechanistically, we propose that the first cysteine residue C126 is the attacking Cys residue that forms a mixed-disulfide complex between TsdA with target proteins; this is then followed by the subsequent attack by the resolving cysteine residue C129 on the intermolecular disulfide, releasing the reduced product and forming a disulfide in the redox enzyme, as seen in DsbA enzymes (30, 36). It is interesting that C126 is needed for the oxidoreductase activities of TsdA in vitro (Fig. 4), whereas in vivo C129 plays an indispensable role in toxin production and pilus assembly (Fig. 5). It is likely that in vivo, the membrane-bound nature of TsdA keeps the enzyme in a catalytically active environment that is different from a redox buffering condition in vitro where the recombinant enzyme, lacking a transmembrane domain, is in a free-floating, diffusible form. It is noteworthy that many bacterial thioredoxin-fold proteins contain serine or threonine in place of either cysteine in the CxxC motif, suggesting that both cysteines in this motif potentially could serve as attacking redox groups (36).

It is important to recognize that the CxxC motif of TsdA is comprised of CPFC, which is somewhat different than that of DsbA [CPHC], MdbA [CPHC], and PDIs [CGHC], all containing a His residue in their CxxC consensus sequences. This positively-charged amino acid is thought to contribute to the high redox potential of these enzymes by a negative charge that forms in the active site after catalysis (10, 37). Since TsdA lacks this His residue, it is likely that TsdA is not primarily an oxidizer but rather a specialized oxidoreductase that is activated under physiological demands. Consistent with this, *tsdA* expression in the wild-type cells is induced at elevated temperature (Fig. 2). This finding also suggests that *tsdA* expression is regulated, perhaps in response to environmental stresses. Thus, it is not surprising that *C. diphtheriae* harbors multiple thiol-disulfide oxidoreductases, as gram-negative bacteria express various thiol-disulfide oxidoreductases that perform oxidative protein folding in various conditions. Future experiments may aim to identify factors, which reoxidize TsdA, as well as MdbA, and regulatory factors, which may control expression of this thiol-disulfide oxidoreductase that potentially safeguards oxidative protein folding in *C. diphtheriae*. Whether this regulation plays a role in the adaptation of Corynebacteria within the host and its pathogenic properties remains an intriguing open question.

## Materials and Methods

### Bacterial Strains, Plasmids, Media, and Cell Growth.

Bacterial strains and plasmids used in this study are listed in *SI Appendix*, Table S3. *C. diphtheriae* strains were grown in heart infusion broth (HIB) or heart infusion agar (HIA) plates at 30 °C or 37 °C. *E. coli* DH5α and S17-1 used for molecular cloning and gene deletions, respectively, were grown in Luria Broth (LB) at 37 °C. Kanamycin (Kan) or ampicillin (Amp) was added at 35 μg/mL or 100 μg/mL when required. Polyclonal antibodies were raised against TsdA in rabbits as previously described (38). DT A antibody (7F2) was purchased from Invitrogen. Reagents were purchased from Sigma unless indicated otherwise.

### Plasmid Construction.

pTsdA* and pT→G. A fragment encompassing the ribosome binding site and coding sequence of *C. diphtheriae tsdA* was PCR-amplified with Phusion ® polymerase using *tsdA*_RBS_F and *tsdA*_R_HindIII (*SI Appendix*, Table S4), and the resulting product was digested with HindIII and 5′ phosphorylated. An arabinose-inducible promoter was PCR-amplified from pBad33 using primers *araC*_F_PstI and *araC*_R (*SI Appendix*, Table S4), and the PCR product was digested with PstI. The two fragments were then ligated into pCGL0243 (*SI Appendix*, Table S3) precut with PstI and HindIII to form pTsdA*. To generate pT→G, a fragment encompassing the promoter region of *tsdA* and its coding sequence was PCR-amplified from genomic DNA isolated from the *C. diphtheriae* suppressor strain S1, using Phusion DNA polymerase (New England Biolabs; NEB). The fragments were cloned into the pCGL0243 vector. The generated plasmids were individually electroporated into the *C. diphtheriae mdbA* mutant strain.

pP^tsdA^-sfGFP and pP^T2G^-sfGFP. The primers PtsdA-HindIII-F and PtsdA-GFP-R (*SI Appendix*, Table S4) were used to PCR-amplify the *tsdA* promoter region genomic DNA obtained from the wild-type *C. diphtheriae* NCTC13129 or suppressor S1, using Phusion DNA polymerase (New England Biolabs; NEB). Similarly, the sfGFP coding sequence was PCR-amplified with pBsk-sfGFP (*SI* *SI Appendix*, Table S3) as template using primers sfGFP-F and sfGFP-BamHI-R (*SI* *SI Appendix*, Table S4). Overlapping PCR was employed to fuse the two generated fragments. The joined fragment was cloned into pCGL0243, and the generated plasmid was electroporated into appropriate *C. diphtheriae* strains.

Recombinant vectors using pMCSG7. To generate a vector expressing a recombinant TsdA protein with a His-tag replacing the N-terminal transmembrane domain, primers H6-TsdA-F and H6-TsdA-R (*SI* *SI Appendix*, Table S4) were used to PCR amplify from the genomic DNA of the *C. diphtheriae* NCTC13129 a fragment coding for residues 33 to 289. The resulting PCR product were cloned into pMCSG7 using ligation-independent cloning as previously reported (39). The resulting plasmid was transformed in *E. coli* L21 (DE3) for protein expression. This plasmid was used to generate recombinant vectors expressing TsdA variants with mutations in the CxxC motif by site-directed mutagenesis (see below).

### Generation of a tsdA Deletion Mutant in C. diphtheria.

A nonpolar, in-frame deletion mutant of *tsdA* was generated using a SacB counterselection method as previously described (40, 41). Briefly, 1-kb fragments up- and downstream of *tsdA* were amplified using appropriate primers (*SI Appendix*, Table S3) and linked together using overlapping PCR. The 2-kb fragment was then cloned into pK19mobsacB (*SI Appendix*, Table S3), and the resulting plasmid was introduced into *E. coli* S17-1 for conjugation with *C. diphtheriae*. Co-integrates resulting from a single crossover event were selected for growth on kanamycin (50 μg/mL) and nalidixic acid (35 μg/mL) plates. Loss of the recombinant plasmid by a second cross-over event resulting in wild-type and mutant alleles was selected for growth on HIA plates containing 10% sucrose. Deletion mutants were identified by PCR and immunoblotting with α-TsdA.

### Site-Directed Mutagenesis.

To construct Cys to Ala or Ser mutations at position 126 and 129 of TsdA, overlapping primers (*SI Appendix*, Table S4) carrying the target mutations were 5′-phosphorylated and used in PCR-amplification using pMCGS7-TsdA or pTsdA* as templates (*SI Appendix*, Table S3) and Phusion DNA polymerase (NEB). The resulting linear plasmids were purified, ligated, and transformed into *E. coli* DH5α. The targeted mutations were confirmed by DNA sequencing.

### Isolation of C. diphtheriae ΔmdbA Suppressor Strains.

Overnight cultures of WT and *C. diphtheriae* Δ*mdbA* grown at 30 °C were diluted 1:50 and shifted to 37 °C for 24 h. Serial dilutions of both strains were plated onto HI agar and incubated overnight at 37 °C. To identify suppressors, plates were screened for Δ*mdbA* colonies similar in size to WT. PCR analysis was performed to confirm the absence of *mdbA* gene in isolated colonies. Suppressor mutations were identified by whole genome sequencing.

### Whole-Genome Sequencing.

Sequencing-ready libraries were constructed with purified genomic DNA of *C. diphtheriae* strains by means of the Nextera DNA sample preparation kit (Illumina). With Nextera technology, the genomic DNA was simultaneously fragmented and tagged with sequencing adapters in a single experimental step. The DNA libraries were sequenced in a 2 × 250 nucleotide paired-end run using a MiSeq reagent kit v2 (500 cycles) and a MiSeq desktop sequencer (Illumina). This shotgun genome sequencing resulted in the following numbers of paired reads: 1,781,258 (control), 1,903,102 (S1), 1,278,266 (S2), and 988,758 (S3). The resulting reads were mapped to the *C. diphtheriae* NCTC 13129 reference genome (GenBank accession number NC_002935.2) using the exact alignment program SARUMAN (Semiglobal Alignment of short Reads Using CUDA and NeedleMAN-Wunsch) (42). Single nucleotide polymorphisms (SNPs) were extracted from the mapped reads by customized Perl scripts using a minimum coverage of ten reads as threshold for detection.

### 5′ Rapid Amplification of cDNA Ends (RACE) PCR.

Identification of the *tsdA* TSS was performed using the Invitrogen 5′ RACE system for rapid amplification of cDNA ends. Briefly, first-strand cDNA was PCR amplified from total mRNA obtained from WT and Δ*mdbA* strains, using primer GSP1-tsdA (*SI Appendix*, Table S4), which annealed at the 3′ end of *tsdA* mRNA, and SuperScript^TM^ II RT, and generated cDNA was purified. Subsequently, a homopolymeric tail was added to the cDNA 3′ end using dCTP and terminal deoxynucleotidyl transferase (TdT). dC-tailed cDNA was PCR-amplified using *Taq* DNA polymerase (Fisher Scientific), with GSP2-tsdA primer and abridged anchor primer (AAP) provided by the manufacturer. This PCR product was diluted (0.1%) and used in a nested PCR reaction using GSP3-tsdA primer and Abridged Universal Amplification Primer (AUAP) provided by the manufacturer, to enrich for specific PCR products. The obtained 5′ RACE PCR products were characterized by DNA sequencing to identify specific *tsdA* TSS(s).

### Reverse Transcriptase and Quantitative Real-Time PCR (qRT PCR).

Log-phase cultures of *C. diphtheriae* grown at 30 °C were normalized to an OD600 of 1.0, two volumes of RNA Protect® Bacteria Reagent (Qiagen) were added, and cells were incubated at room temperature for 5 min. Then cells were collected by centrifugation, washed once with PBS, resuspended in RLT buffer (RNeasy Mini Kit, Qiagen) containing β-marcaptoethanol (BME) and lysed by mechanical disruption with 0.1-mm silica spheres (MP Bio) in a ribolyser (Hybaid). Total RNA from cell lysates was extracted using the RNAeasy Mini Kit (Qiagen). Purified total RNA was treated with DNAse I to digest remaining DNA. After the enzymatic reaction, RNA was cleaned using the RNeasy MinElute Cleanup Kit (Qiagen). cDNA was synthesized with SuperScript^TM^ II RT First-Strand Synthesis System (Invitrogen). For qRT-PCRs cDNA was mixed with iTAQ SYBR green supermix (Bio-Rad), along with appropriate primer sets (*SI Appendix*, Table S4). Cycle threshold (*C_T_*) values were determined, and the 16S rRNA gene was used as a control to calculate relative mRNA expression level by the 2^−ΔΔ^*CT* method (43).

### Cell Fractionation and Western Blotting.

For DT production, overnight cultures of *C. diphtheriae* were used to inoculate fresh cultures, which were grown at 30 °C until OD_600_ reached between 0.2 and 0.3. Iron chelator ethylenediamine-di-(o-hydroxyphenylacetic) acid (EDDA) was added to the cultures at the concentration of 0.05 mg/mL. After 5 h of inoculation at 30 °C, the culture supernatant was collected for TCA precipitation and acetone wash.

For pilus assembly and membrane protein detection, corynebacterial cells were grown at 30 °C to mid-log phase, normalized to an OD_600_ of 1.0, and subjected to cell fractionation as previous reported (15). Protein samples collected from the culture supernatant (S), cell wall (W), and membrane (M) fractions were analyzed by immunoblotting with specific antibodies (α-SpaA, 1:20,000 dilution; α-DT, 1:1,000; α-TsdA, 1:5,000; α-MdbA, 1:5,000).

### Protein Purification.

His-tagged MdbA or TsdA proteins were purified according to a published procedure with some modification (18). Briefly, *E. coli* BL21 (DE3) cells harboring recombinant plasmids were cultured in LB medium supplemented with ampicillin (100 µg/mL) at 37 °C. When cells reached OD_600_ of 0.8, isopropyl β-D-1-thiogalactopyranoside (IPTG) was added to a final concentration of 0.5 mM for overnight induction at 18 °C. Cells were harvested by centrifugation and lysed by sonication, and clear lysates were obtained by centrifugation. Recombinant proteins were purified by affinity chromatography using a Ni-NTA epharose column (Qiagen), followed by an Econo-Pac 10DG column (Bio-Rad) in 100 mM potassium acetate buffer, pH 7.5 and stored at −20 °C for further experimentation.

For crystallization, purified TsdA proteins were digested with 0.15 mg TEV protease per 20 mg of purified protein for 16 h at 4 °C, and then passed through a Ni-NTA column to remove both the TEV protease and cleaved N-terminal tags. The final step of purification was gel-filtration on HiLoad 16/60 Superdex 200 pg column (GE Healthcare) in 10 mM HEPES buffer pH 7.5, 200 mM NaCl and 1 mM DTT. The protein was concentrated on Amicon Ultracel 10K centrifugal filters (Millipore) up to 60 mg/mL concentration.

### TsdA Structure Determination.

The initial crystallization condition was determined with a sparse crystallization matrix at 4 °C and 16 °C temperatures using the sitting-drop vapor-diffusion technique using MCSG crystallization suite (Microlytic), Pi-minimal and Pi-PEG screen (44) (Jena Bioscience). The best crystals were obtained from F2 conditions of Pi-PEG screen (6.4% PEG 200, 21.4% PEG 2000, 50 mM acetate buffer pH 5.2) at 4 °C temperature. Crystals selected for data collection were briefly soaked in crystallization buffer with addition of 20% ethylene glycol as cryo-protectant and then flash-cooled in liquid nitrogen.

Single-wavelength X-ray diffraction data were collected at 100 K temperature at the 19-ID and 19BM beamlines of the Structural Biology Center (45) at the Advanced Photon Source at Argonne National Laboratory using the program SBC collect. The intensities were integrated and scaled with the HKL3000 suite (46). The wild-type and TsdA-C129S structures were determined by single-wavelength anomalous dispersion (SAD) phasing and molecular replacement, respectively, using HKL3000 suite (46). Several rounds of manual adjustments of structure models using COOT (47) and anisotropic refinements with Refmac program (48) from CCP4 suite (49) were done. The stereochemistry of the structure was validated with PHENIX suite (50) incorporating MOLPROBITY (51) tools. The secondary structure assignment was generated by DSSP program (52) incorporated in ESPRIPT (53) server. A summary of data collection and refinement statistics are presented in *SI Appendix*, Tables S1 and S2. The atomic coordinates and structure factors of WT TsdA and its C129S mutant have been deposited in the Protein Data Bank, www.wwpdb.org (PDB ID codes 7UNN and 7UNO, respectively).

### Fluorescence Microscopy.

To characterize *tsdA* transcriptional regulation, expression of *tsdA* was measured using a sfGFP transcriptional reporter. Overnight cultures of *C. diphtheriae* strains harboring individual sfGFP transcriptional reporters were diluted and grown at 37 °C until reaching mid-log phase; at this point a subset of bacterial cultures were inoculated at 40 °C for 30 min. To quantify fluorescence signal, cells harvested by centrifugation were washed with PBS and normalized to OD_600_ of 0.5. Cell suspension aliquots were dispensed into 96-well, high-binding, clear F-Bottom (Chimney well), black microplates (Greiner bio-one). Fluorescence was measured using excitation/emission wavelengths 485 nm/507 nm with a Tecan M1000 plate reader. Purified sfGFP was used as gain reference and the fluorescence of wild-type strain carrying no plasmid was used as the background signal.

In a parallel experiment, cells were then placed on agarose (1.5%) pads and viewed by an Olympus IX81- ZDC inverted fluorescence microscope using excitation/emission wavelengths 504 nm/510 nm.

### Insulin Reduction Assay.

The reductase activity assay was adapted from previously established protocol (54). Briefly, insulin (MP Biomedicals) was prepared at 20 mg/mL in 0.02 N HCl. The assay was carried out in a 96-well plate containing 100 mM potassium acetate buffer pH 7.5, 0.5 mM DTT, 2 mM EDTA, 170 µM insulin, and 30 µM purified enzymes (MdbA or TsdA) at 25 °C, for 80 min. The turbidity of reduced insulin was monitored at 650 nm, for every 4 min using a Tecan M1000 plate reader, reactions without enzymes addition used as control. The data, obtained from triplicate experiments, were plotted with subtraction from blank samples in the absence of DTT.

### Dsb Reconstitution of FimA.

Dsb formation of FimA was performed as previously described with some modification(18). Briefly, 3 µM DTT-treated FimA (reduced FimA) was incubated with 25 µM purified TsdA proteins in a redox buffer containing 100 mm Tris-HCl, pH 4.0, 2 mm EDTA, 0.2 mm GSSG, 1 mm GSH. After 30 min incubation at 37 °C, one volume of alkylation buffer (20 mm Mal-PEG, 2% SDS, 200 mm Tris-HCl, pH 8.0) was added to stop the reactions. All samples were then slightly rotated at 37 °C for 1 h in the dark. The alkylated samples were TCA-precipitated, suspended in SDS-loading buffer, analyzed by SDS-PAGE with Coomassie staining.

### Statistical Analysis.

Results are presented as averages from three independent experiments and SD. Statistical analysis was performed by unpaired *t* test with Welch’s correction determined using GraphPad Prism 5.0 (La Jolla, CA).

## Supplementary Material

Appendix 01 (PDF)

## Data Availability

All study data are included in the article and/or *SI Appendix*.
