# Supplementary material for: A cryptic oxidoreductase safeguards oxidative protein folding in Corynebacterium diphtheriae
Source: Proc Natl Acad Sci U S A. 2023 Feb 14;120(8):e2208675120. doi: 10.1073/pnas.2208675120 (PMC9974433; doi:10.1073/pnas.2208675120)
Supplement: Supplementary file 1 — Appendix 01 (PDF) [file pnas.2208675120.sapp.pdf]

A cryptic oxidoreductase safeguards oxidative protein folding in *Corynebacterium diphtheriae*

Melissa E. Reardon-Robinson[1*], Minh Tan Nguyen[2*], Belkys C. Sanchez[1,3*], Jerzy Osipiuk[4,5], Christian Rückert[6], Chungyu Chang[2], Bo Chen[1], Rahul Nagvekar[1,7], Andrzej Joachimiak[4,5], Andreas Tauch[6], Asis Das[8], and Hung Ton-That[2,9,10†]

[1]*Department of Microbiology & Molecular Genetics, University of Texas McGovern Medical School, Houston, Texas, USA;* [2]*Division of Oral Biology and Medicine, School of Dentistry, University of California, Los Angeles, California, USA;* [3]*Department of Molecular Virology and Microbiology, Baylor College of Medicine, Houston, TX, USA;* [4]*Center for Structural Genomics of Infectious Diseases (CSGID), Consortium for Advanced Science and Engineering, University of Chicago, Chicago, IL, USA;* [5]*Structural Biology Center, Argonne National Laboratory, Lemont, IL, USA;* [6]*Center for Biotechnology (CeBiTec), Bielefeld University, Bielefeld, Germany;* [7]*Stanford University, Stanford, CA, USA;* [8]*Department of Medicine, Neag Comprehensive Cancer Center, University of Connecticut Health Center, Farmington, CT, USA;* [9]*Molecular Biology Institute, University of California, Los Angeles, California, USA;* [10]*Department of Microbiology, Immunology & Molecular Genetics, University of California, Los Angeles, Los Angeles, CA, USA*

*Equal contribution

†To whom correspondence may be addressed:

Hung Ton-That, htonthat@dentistry.ucla.edu

Running Title: *Compensatory thiol-disulfide oxidoreductase in C. diphtheriae*

Keywords: *Corynebacterium diphtheriae*, disulfide bond, pili, diphtheria toxin, Gram-positive bacteria

Supporting Figures

Figure S1: Normal cell morphology of *C. diphtheriae* Δ*mdbA* suppressor mutants at the non-permissive temperature. (A-F) Stationary cultures of indicated *C. diphtheriae* strains grown at 30°C were diluted into fresh media and incubated at 30°C or 37°C until the wild-type cells reached log phase. Harvested corynebacterial cells were analyzed by electron microscopy via negative staining with 1% uranyl acetate. Scale bars indicate 0.5μm.

Figure S2: 5' RACE analysis for the transcriptional start site of *tsdA*. mRNA of the wild-type and suppressor S1 strains was subjected to 5' RACE analysis, resulting in a homopolymeric tail added to the 3' end of the cDNA. DNA chromatograms from the sequencing reactions of 5' RACE PCR products are shown, with the transcriptional direction indicated by arrows.

Figure S3: Stationary cultures of indicated *C. diphtheriae* strains grown at 30°C were diluted into fresh media and incubated at 30°C or 37°C until cells reached log phase and then were subjected to cell fractionation. Protein samples in culture supernatant (M), and cell wall (M) fractions were immunoblotted with α-SpaA. Monomers (M) and polymers (P) of SpaA and molecular weight markers are indicated.

Figure S4. Structure alignments of *C. diphtheriae* TsdA with other oxidoreductases. The *C. diphtheriae* TsdA structure (green) was aligned with 4PWO (gray) **(A)**, *E. coli* DsbA (salmon; PDB:6BQX) **(B)**, and *Bacillus subtilis* BdbD (orange; PDB:3EU3) **(C)**.

Figure S5: Purification of recombinant thiol-disulfide oxidoreductase proteins. Recombinant thiol-disulfide oxidoreductase proteins were expressed and purified from *E. coli*, using affinity chromatography. Purified proteins were analyzed by SDS-PAGE and Coomassie staining.

Supporting Tables

Table S1: Data processing [a]

**Structure**	**TsdA**	**TsdA-C129S**
Space group	$P\ 2_1 2_1 2_1$	$P\ 2_1 2_1 2_1$
Unit cell dimensions	a = 52.2 Å, b = 65.4 Å,<br>c = 68.9 Å, α = β = γ = 90°	a = 52.8 Å, b = 65.1 Å,<br>c = 69.4 Å, α = β = γ = 90°
Resolution range (Å)	30.5-1.45 (1.48–1.45)	47.5 - 1.10 (1.12 - 1.10)
No. of unique reflections	42,523 (1,851)	95,848 (3,908)
Completeness	99.4 (89.6)	98.2 (80.6)
R-merge	0.084 (0.95)	0.065 (0.80)
CC1/2 (Å^2)	0.992 (0.45)	0.997 (0.46)
I/σ	25.3 (1.09)	40.1 (1.25)
Redundancy	6.3 (2.8)	7.6 (2.2)
Wilson B-factor (Å^2)	14.0	9.6

[a] Values in parentheses correspond to the highest resolution shell.

Table S2: Structure refinement statistics

Structure	TsdA	TsdA-C129S
Refinement		
Resolution range (Å)	30.5-1.45	47.5-1.10
Reflections work/test	40,362/2,097	91,039/4,730
R_{work}/R_{free}	0.114/0.153	0.116 /0.134
RMSD (bonds) (Å)	0.010	0.010
RMSD (angles) (°)	1.59	1.59
Number of atoms		
protein	1984	2067
Ligand/ion	17	15
Water	292	416
B-factors		
Average B-factor ($Å^2$)	21.1	16.9
Protein	19.0	14.0
Ligand/ion	31.9	30.0
Water	34.9	30.5
MolProbity validation		
Ramachandran outliers (%)	0.00	0.0
Ramachandran favored (%)	97.9	98.8
Rotamer outliers (%)	0.46	0.0
Clashscore	1.49	0.71
MolProbity score	0.90	0.73
PDB ID	7UNN	7UNO

Table S3: Strains and Plasmids used in this study

Strains & Plasmids	Description	Reference
Strain		
NCTC13129	Wild-type	(1)
NJ1	Isogenic derivative of NCTC13129; Δ*mdbA*	(2)
HT1	Isogenic derivative of NCTC13129; Δ*tox*	(2)
MR1	Derivative of NJ1; Δ*mdbA* suppressor 1 (S1)	This study
MR2	Derivative of NJ1; Δ*mdbA* suppressor 2 (S2)	This study
MR3	Derivative of NJ1; Δ*mdbA* suppressor 3 (S3)	This study
MR4	Isogenic derivative of NCTC13129; Δ*tsdA*	This study
MR5	NJ1 containing pT→G	This study
MR6	NJ1 containing pTsdA	This study
BCS1	MR4 containing pP^{tsdA}-sfGFP	This study
BCS2	MR4 containing $pP^{\textit{tsdA}\text{-T2G}}$-sfGFP	This study
MTN1	NJ1 containing pCGL243-AraC-TsdA	This study
MTN2	NJ1 containingpCGL243-AraC-TsdA-C126S	This study
MTN3	NJ1 containing pCGL243-AraC-TsdA-C126A	This study
MTN4	NJ1 containing pCGL243-AraC-TsdA-C129S	This study
MTN5	NJ1 containing pCGL243-AraC-TsdA-C129A	This study
Plasmids		
pCGL0243	*Corynebacterium/E. coli* shuttle vector; kanamycin resistant	(3)
pBad33	Vector containing the arabinose P_{BAD} promoter	(4)
pK19mobsacB	*Corynebacterium* integration plasmid; kanamycin resistant	(5)
pMCSG7	Expression vector; ampicillin resistant	(6)
pK19mobsacB-TsdA	pK19mobsacB allelic replacement of *tsdA*	This study
pT→G	pCGL0243 expressing *tsdA* from its promoter in suppressor S1	This study
pTsdA*	pCGL0243 expressing TsdA under control of AraC	This study
pC126S	Derivative of pTsdA* expressing TsdA-C126S	This study
pC126A	Derivative of pTsdA* expressing TsdA-C126A	This study
pC129S	Derivative of pTsdA* expressing TsdA-C129S	This study
pC129A	Derivative of pTsdA* expressing TsdA-C129A	This study
pMCSG7-MdbA	For expression of recombinant MdbA	(2)
pMCSG7-TsdA	For expression of recombinant TsdA	This study
pMCSG7-TsdA-C126S	For expression of recombinant TsdA harboring C126S mutation	This study
pMCSG7-TsdA-C126A	For expression of recombinant TsdA harboring C126A mutation	This study
pMCSG7-TsdA-C129S	For expression of recombinant TsdA harboring C129S mutation	This study
pMCSG7-TsdA-C129A	For expression of recombinant TsdA harboring C129A mutation	This study
pBsk-sfGFP	sfGFP expressing plasmid	(7)

pP^{tsdA}-sfGFP	pCGL0243 expressing sfGFP from the wild-type *tsdA* promoter	This study
pP$^{tsdA\text{-T2G}}$-sfGFP	pCGL0243 expressing sfGFP from the T2G *tsdA* mutant promoter	This study

Table S4: Primers used in this study

Primer	Sequence[(a)(b)]	Application
tsdA-A-XbaI	AAATCTAGAGTTTGAGGAAAGCGGTTT	pK19mobsacB-TsdA
tsdA-B-R	CCCATCCACTAAACTTAAACACTTCTGCATGAAGTACAT	pK19mobsacB-TsdA
tsdA-C-F	TGTTTAAGTTTAGTGGATGGGTGATTGAGTCGGAGCTGA	pK19mobsacB-TsdA
tsdA-D-XbaI	AAATCTAGAGAACTCGCCGCCAGCGAA	pK19mobsacB-TsdA
GSP1-*tsdA*	GTTAGACCAGCGGGCACAGAA	5’ RACE
GSP2-*tsdA*	GGGGCACTCGAAGTCAGAGAA	5’ RACE
GSP3-*tsdA*	TTCCGTAATCACCAGCGGTGC	5’ RACE
RTPCR-*tsdA*-F	TAGCGGTAAGGCGGGTTCG	RT-PCR
RTPCR-*tsdA*-R	GATCTTTCGCGTTACGACGGTG	RT-PCR
tsdA-RBS-F	AAGGATCCAGAGTGTCTCGTTGGGTCGC	pTsdA*
tsdA-R-HindIII	AA AAGCTTTTAGTTTTGGTGGCTGGAAG	pTsdA*
araC-F-PstI	AAACTGCAGTTATGACAACTTGACGGCTACATCATTCAC	pTsdA*
araC-R	TACCAATTATGACAACTTGAC	pTsdA*
H6-TsdA-F	TACTTCCAATCCAATGCAAATAAGTCGGCAGGCACCCAAG	pMCSG7-TsdA
H6-TsdA-R	TTATCCACTTCCAATGTTAGTTTTGGTGGCTGGAAGGCG	pMCSG7-TsdA
TsdA-C126A/S-F	CCCTTCTGTGCCCGCTGGTCTAAC	Site-directed mutagenesis
TsdA-C126S-R	**GCT**CTCGAAGTCAGAGAATTCCGTAATCACCAGC	Site-directed mutagenesis
TsdA-C126A-R	**CGC**CTCGAAGTCAGAGAATTCCGTAATCACCAG	Site-directed mutagenesis
TsdA-C129S-F	**AGC**GCCCGCTGGTCTAACCAGACCGAG	Site-directed mutagenesis
TsdA-C129A-F	**GCG**GCCCGCTGGTCTAACCAGACCG	Site-directed mutagenesis
TsdA-C129A/S-R	GAAGGGGCACTCGAAGTCAGAGAATTC	Site-directed mutagenesis
PtsdA-HindIII-F	AAAAAAAGCTTCGTAGAAAACTCGGTAAGTAAGCC	pP^{tsdA}-sfGFP
PtsdA-GFP-R	CGCTGACTTCTGCATGAAGTACATATGTCTAAAGGTGAAGAACTGTTC	pP^{tsdA}-sfGFP reporter
sfGFP-F	ATGTCTAAAGGTGAAGAACTGTTC	pP^{tsdA}-sfGFP reporter
sfGFP-BamHI-R	AAAAAGGATCCCTATTTGTAGAGCTCATCCATGCC	pP^{tsdA}-sfGFP reporter

[a] Underlined are restriction site sequences.

[b] Bold are mutation site sequences.

References

1. Ton-That H & Schneewind O (2003) Assembly of pili on the surface of *Corynebacterium diphtheriae*. *Mol Microbiol* 50(4):1429-1438.

2. Reardon-Robinson ME*, et al.* (2015) A thiol-disulfide oxidoreductase of the Gram-positive pathogen *Corynebacterium diphtheriae* is essential for viability, pilus assembly, toxin production and virulence. *Mol Microbiol* 98(6):1037-1050.

3. Ankri S, Reyes O, & Leblon G (1996) Electrotransformation of highly DNA-restrictive corynebacteria with synthetic DNA. *Plasmid* 35(1):62-66.

4. Guzman LM, Belin D, Carson MJ, & Beckwith J (1995) Tight regulation, modulation, and high-level expression by vectors containing the arabinose PBAD promoter. *J Bacteriol* 177(14):4121-4130.

5. Reyes O*, et al.* (1991) 'Integron'-bearing vectors: a method suitable for stable chromosomal integration in highly restrictive corynebacteria. *Gene* 107(1):61-68.

6. Stols L*, et al.* (2002) A new vector for high-throughput, ligation-independent cloning encoding a tobacco etch virus protease cleavage site. *Protein Expr Purif* 25(1):8-15.

7. Rohrschneider LR, Custodio JM, Anderson TA, Miller CP, & Gu H (2005) The intron 5/6 promoter region of the ship1 gene regulates expression in stem/progenitor cells of the mouse embryo. *Dev Biol* 283(2):503-521.

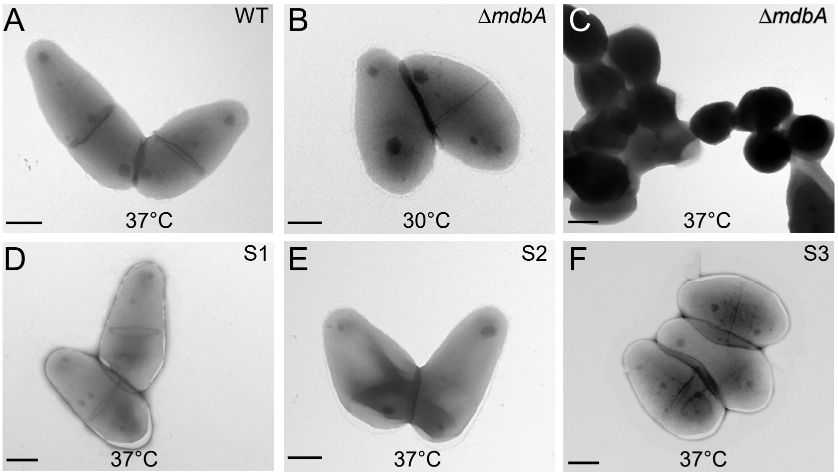

Figure S1: Robinson-Reardon et al.

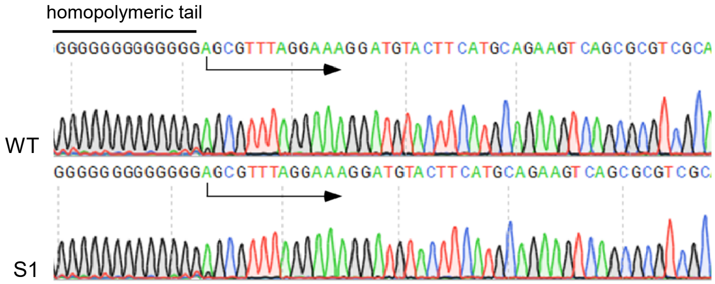

Figure S2: Reardon-Robinson et al.

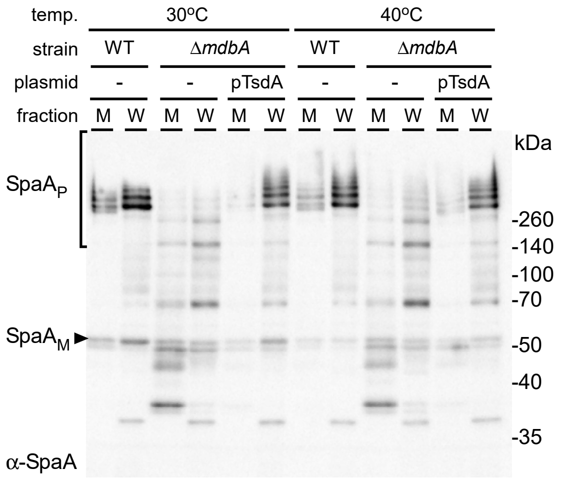

Figure S3: Reardon-Robinson et al.

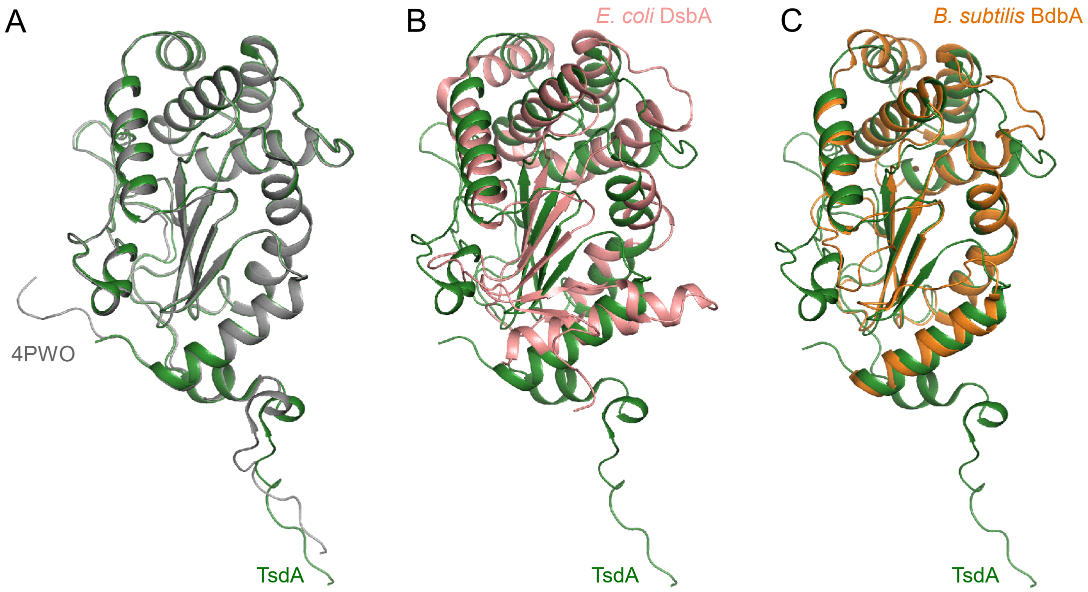

Figure S4: Reardon-Robinson et al.

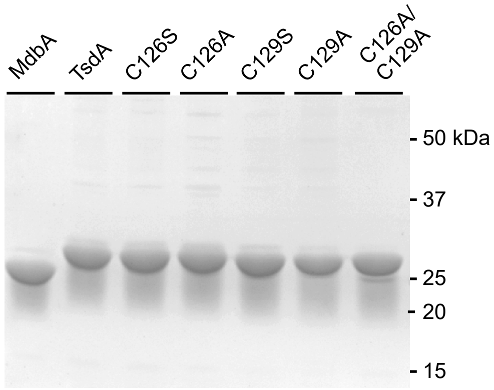

Figure S5: Reardon-Robinson et al.
